# Integrative health care – What are the relevant health outcomes from a practice perspective? A survey

**DOI:** 10.1186/s12906-017-2041-4

**Published:** 2017-12-22

**Authors:** Ania Kania-Richmond, Amy Metcalfe

**Affiliations:** 1 Department of Community Health Science, Cumming School of Medicine, University of Calgary, Calgary, AB Canada; 2 Department of Obstetrics and Gynecology, Cumming School of Medicine, University of Calgary, Calgary, AB Canada

**Keywords:** Outcomes, Health domains, Integrative health care, Integrative medicine

## Abstract

**Background:**

Integrative health care (IHC) is an innovative approach to health care delivery. There is increasing focus on and demand for the evaluation of IHC practices. To ensure such evaluations capture their full scope, a clear understanding of the types of outcomes relevant to an IHC approach is needed. The objective was to describe the health domains and health outcomes relevant to IHC practices in Canada.

**Methods:**

An online survey of Canadian IHC clinics. Survey questions were informed by the IN-CAM Health Outcomes Database. Descriptive statistics were used to summarize the data. Chi square tests were used to compare responses between clinic types and patient groups served.

**Results:**

Surveys were completed by 21 clinics (response rate: 50%). Physical, psychological, social, individualized and holistic were identified as applicable health domains by more than 90% of the clinics. Spiritual domain was the least relevant (70% of clinics). A number of relevant outcomes within each domain were identified. A core set of outcomes were identified and included: fatigue, anxiety, stress, and patient-provider relationship, and quality of life. Clinics with primarily conventional health practitioners were less likely to address overall well-being (*p* = 0.04), while clinics that provided care to a specialized patient population (i.e. cancer patients) or a mix of general and specialized patients were less likely to address religious practices (*p* = 0.04) or spiritual experiences (*p* = 0.007).

**Conclusions:**

Outcomes across health domains should be considered in the evaluation of IHC models to generate an understanding of the full scope of effectiveness of IHC approaches. The core set of outcomes identified may facilitate this task.

Ethics approval (Ethics ID REB14-0495) was received from the Conjoint Health Research Ethics Board at the University of Calgary.

**Electronic supplementary material:**

The online version of this article (10.1186/s12906-017-2041-4) contains supplementary material, which is available to authorized users.

## Background

Integrative health care (IHC) practices are an approach to care that has emerged in response to a number of factors including patient demand for complementary therapies, their use of complementary and conventional treatments simultaneously, challenges associated with chronic disease management, and increasing focus on disease prevention and health promotion. This has resulted in increasing IHC-specific research activity, development and implementation of IHC practices and programs, and health professional educational programming in this area [1, 2].

There is general consensus that IHC is a distinct approach to care, which involves bringing together complementary and conventional treatment approaches in a coordinated manner to address an individual’s health needs [3]. However, as reflected in the varied definitions proposed in the literature, and processes and structures of IHC practices, it remains a concept that means different things to different people [4–7]. Further, integration may occur at the level of the practitioner, team, clinic, or health system [8]. Drawing on the literature [5, 9–12], we define IHC as an approach to care that brings together CAM and conventional approaches, and is based on a philosophy of care that encompasses the following four components: 1) whole person/holistic perspective, 2) personalized or individualized care, 3) patient centeredness, and 4) a focus on wellness. Descriptions of each component are provided in Table 1.

**Table 1:** Descriptions of core components of IHC

Component	Description
1) Whole person care/holistic	The person being treated is regarded as an integrated combination of multiple dimensions, which include the physical body, the mind, the spiritual element of one’s beliefs, and context (social/relationships, environment)
2) Personalized or individualized care	Treatment is specifically tailored to address the patient’s unique constitution and needs
3) Patient centeredness	Patient is directly involved in the decision making process regarding their care and treatment options
4) Wellness	Emphasis or focus is placed on enhancing and supporting health and well-being and preventing disease.

Although increasingly popular, the evidence for the IHC approach is growing but limited [5, 13]. Assessment of such practices, both through formal research and practice-based evaluations, is critical in understanding benefits, improving clinical outcomes and advancing the practice of IHC. However, there are challenges in this area of inquiry. One is the heterogeneity of the IHC approach. Another is consideration of the contextual factors that impact how an intervention is delivered and its potential therapeutic benefit: processes and structures including assessment, clinical expertise, and professional collaborations [14–16]. And of course, there is the question of health outcomes: what should or needs to be assessed to determine efficacy, effectiveness and safety and how this is achieved. Further, how can the numerous variables within an individualized care plan be accommodated, which may also change over the course of a treatment plan [13].

Understanding outcomes relevant to and reflective of a given health care approach, here it being IHC, and identifying valid and reliable measures of those outcomes, is critical in our ability to effectively assess relevant outcomes, compare across settings or studies, and also to support optimal clinical decision making [17]. Yet, this is challenging. In studying IHC, researchers have pre-dominantly applied tools developed within the conventional care context [18, 19]. This has limitations in effectively capturing the full picture of purported IHC outcomes. For example, applying the standardized quality of life measures (SF-36) limits the scope to pre-selected functional aspects of physical and emotional health but omits aspects such as spirituality [15, 17, 18]. Additionally, outcomes of interest in most IHC research are selected to capture a specific symptoms or changes in a disease state/process under study. However, this does not align fully with the underlying IHC philosophy of care nor does it capture the full scope of potential benefit gained through an IHC approach. This disconnect also exists as questions addressed through research are most often determined by the researchers’ interests and/or research program. To date, there has been limited consideration of what outcomes are relevant to clinicians, and their patients, in IHC settings [20]. And yet it is the patient demand and the emergence of these practices that is driving the research activity.

The purpose of this study is to identify health outcomes considered to be relevant within established IHC practices in Canada to inform assessments of treatment progress and evaluate this innovative approach to care delivery in a more responsive and comprehensive manner.

## Methods

### Clinic sample

Our sample of IHC clinics, centers and/or practices was identified through a rigorous internet search (search engines: Google and Yahoo), applying various combinations of the following keywords: province name (British Columbia or Alberta or Saskatchewan Manitoba or Ontario or Quebec or New Brunswick or Nova Scotia or Prince Edward Island or Newfoundland) AND integrat* AND health care or medicine OR complementary health care or medicine OR holistic health care or medicine AND clinic or center or centre or practice. Based on the definition of IHC applied in this study and the requirement for an online presence (to enable access), our inclusion criteria were: clinics that self-identified as “integrative”; had a website presence (active website) and email address; and the following concepts were presented on the clinic website as part of the clinic’s approach, mission, vision and/or goals: holistic approach care, provision of what is currently classified as complementary and conventional therapy services, services provided by more than one practitioner (team-based/multi-professional), individualized care, and disease prevention/health promotion. The sample was supplemented through requested input from researchers and health care practitioners who were members of the IN-CAM Research Network (www.incamresearch.ca).

### Survey instrument

The survey was developed for the purposes of this study. The IN-CAM Health Outcomes Database (the Database) [21] was used to inform survey questions specific to health domains and health outcomes. The Database was developed in order to identify outcome measures of particular importance to complementary and integrative health care effectiveness and efficacy research and to assist researchers and practitioners to frame their approach within an integrated perspective.

The focus of this survey was on clinical health outcomes, which are the change in symptomatology or health status of an individual, group or population that are attributable to a planned and delivered health intervention [22]. With patient centered care a core component of the IHC approach, patient reported outcomes (PROs) [23] were of interest. Health domains are groupings of health outcomes to relate to an area of human health (e.g. psychological, physical, spiritual) [21].

The survey was reviewed and face validity was established through consultation with five experts in the field. The final survey consisted of 22 questions. Three of the questions were consent based, as per ethical requirements. Five questions addressed clinic and practitioner information - location, name of clinic (optional), and individual’s position at the clinic, contact information (optional). One question addressed the concepts used to define IHC, and one question provided an open field for additional comments. Two questions were related to survey follow up. The remaining ten questions focused on health outcomes within five health domains (physical, psychological, social, spiritual, global/holistic and individualized) and outcomes measurement. The final survey is provided in Additional file 1.

### Recruitment and data collection

The initial phase of recruitment and data collection took place between September and November 2014, with a second round of recruitment and data collection between May and July 2015. All identified clinics were contacted to introduce the clinic to the study, gauge their interest in participation, and identify the most appropriate person to complete the survey. The identified individual was then emailed a link to the survey, along with up to three reminder emails over a period of 4 weeks. All surveys were completed online via SurveyMonkey and raw data was collected through the SurveyMonkey server.

### Analysis

Descriptive statistics were used to characterize the clinics, summarize the health domains and outcomes, and how outcomes were captured or measured in the practice. Chi square tests were used to compare differences in the relevancy of the elements used to define integrative health care and the health domains and outcomes by a) practitioner type mix (primarily conventional, mainly CAM, and equally mixed practices) and b) type of practice (generalized, specialized, and mixed practices). Alpha <0.05 was deemed statistically significant. All analyses were conducted using Stata SE version 13.

## Results

The initial internet based search conducted by AKR and HR, with input from IN-CAM researchers and clinicians, resulted in identification of 204 clinics that self-identified as providing an “integrative health care” approach. Of these, 42 clinics met our inclusion criteria. All were included, individually contacted, and invited in participate in the survey. The survey was completed by 21 clinics (response rate: 50%).

Locations of the 21 clinics were: Alberta (8), Ontario (7), British Columbia (5) and Quebec (1). The surveys were completed by clinic owners (11), directors (9), practitioners (8), founders (6) or administrative staff (4).

All participating clinics were asked to rate how relevant each IHC component (Table 1) was to their clinic, on a scale from very relevant to not relevant; “do not know” was also a response option provided. All five components were relevant to almost all of the clinics. Personalized or individualized care was *very relevant* for all clinics. Whole person care and patient centered care were considered *very relevant* by 20 out of 21 clinics (95%) and somewhat relevant by 1 clinic (5%). Combining CAM and conventional practices was *very relevant* to 19 out of 21 clinics (90%), s*omewhat relevant* for one clinic (5%), and one clinic (5%) indicated a *neutral* position. Wellness and disease prevention was *very relevant* to 17 out of 21 clinics (80%); 3 clinics (15%) indicated it was *somewhat relevant* and one clinic indicated a *neutral* position.

**Fig. 1:**
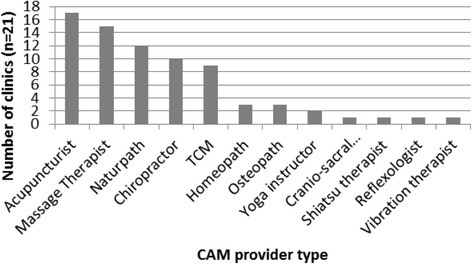
Number of study sites providing services delivered by CAM providers

**Fig. 2:**
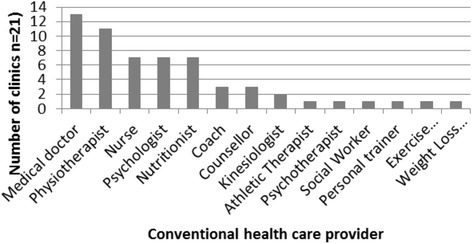
Number of study sites providing services delivered by conventional health care providers

Health care services were delivered by a broad range of health care practitioners across the clinics. Twenty-six different practitioner were identified, consisting of 12 different types of CAM practitioners (see Fig. 1) and 14 types of conventional health care practitioners (see Fig. 2).

The most common types of practitioners were: acupuncturists (17 clinics; 81%), followed by massage therapists (15 clinics; 71%), medical doctors (13 clinics; 62%), naturopathic doctors (12 clinics; 57%), physiotherapists (11 clinics; 52%), chiropractors (10 clinics; 47%), and traditional Chinese medicine (TCM) practitioners (9 clinics, 42%). The number of practitioner types per clinic ranged from 2 to 11, with an average of 6.5 types per clinic (see Table 2). The mix of practitioner types was highly variable across the sites.

**Table 2:** Practitioner type mix across study sites

Practitioner type	Study site:																				
CAM	1	2	3	4	5	6	7	8	9	10	11	12	13	14	15	16	17	18	19	20	21
Acupuncturist	X	X	X	X	X	X	X				X	X	X	X	X	X		X	X	X	X
Massage Therapist		X	X	X		X	X	X		X		X			X	X	X	X	X	X	X
Naturpath	X			X			X		X			X	X		X	X	X	X	X	X	
Chiropractor				X		X	X	X		X		X			X	X	X	X			
TCM		X	X	X	X							X		X				X	X	X	
Homeopath		X			X		X														
Osteopath		X										X	X								
Yoga instructor			X																	X	
Cranio-sacral															X						
Shiatsu therapist															X						
Reflexologist																				X	
Vibration therapist																					X
Conventional	1	2	3	4	5	6	7	8	9	10	11	12	13	14	15	16	17	18	19	20	21
Medical doctor	X		X	X		X			X		X	X	X	X	X	X			X	X	
Physiotherapist				X		X		X		X					X	X	X	X	X	X	X
Nurse	X			X								X			X	X			X	X	
Psychologist		X	X	X	X								X	X						X	
Nutritionist	X									X	X	X	X			X				X	
Coach			X													X					X
Counsellor							X				X	X									
Kinesiologist						X					X										
Athletic Therapist																					X
Psychotherapist															X						
Social Worker																	x				
Personal trainer																X					
Exercise Physiologist																X					
Weight Loss consultant				X																	
Hypnotherapist							X														
Number of practitioners types:	5	6	7	10	4	6	7	3	2	4	5	10	6	4	10	11	5	6	7	11	6
^a^Balance of practitioner types:	C	CA	CA	E	CA	E	CA	CA	E	E	C	CA	E	E	CA	CO	CA	CA	CA	CA	E

^a^C = higher number of conventional practitioners; CA = higher number of CAM practitioners; E = equal number of conventional and CAM practitioners

Clinics were placed into one of three categorized based on the type of practitioner present and number of each type: 1) CAM-oriented clinics (more types of CAM than conventional practitioners), 2) Conventionally-oriented (more types of conventional than CAM practitioners), and 3) Equal mix (same number of CAM and conventional practitioner types). CAM oriented clinics were most common (12 clinics; 57%), followed by equal mix clinics (6 clinics; 29%). Clinics where there were more types of conventional than CAM practitioners were least common (3 clinics; 14%) (see Table 2).

The general population had access to care at 16 of the clinics. Eighteen clinics provided health services to specific or specialized patient populations, including performers/artists (1), individuals being supported through a company sponsored health and wellness (1), and patients with cancer (3), mental health needs (1), injuries sustained from a motor vehicle accident (1), or catastrophic impairments (1).

All clinics identified the physical and psychological *health domains* as relevant or important in relation to the care provided. Overall or global well-being and individualized outcomes were relevant to all but one clinic. The social domain was relevant for 19/21 clinics (90%). Spirituality was the least relevant, identified by 15/21 clinics (70%).

The *health outcomes* identified as relevant for each health domain are presented in Table 3. The most common outcomes in the physical domain were fatigue, sleep quality and physical function (mobility, impairment). In the psychological domain, they included anxiety, stress, pain and depression. In the spiritual domain, these were peace, spiritual wellbeing, meaning of life, serenity. And lastly, in the holistic domain, these were quality of life, overall well-being, and health status. Spirituality-oriented outcomes were indicated as not applicable at 4/21 clinics (40%). The health outcomes identified as relevant or important across all study sites were fatigue (physical domain), anxiety and stress (both in the psychological domain), the patient-provider relationship (social domain) and quality of life (holistic domain).

**Table 3:** Outcomes within each health domain identified as relevant and/or important across the study sites

	Health domin:					
	Physical	Psychological	Social	Spiritual	Global or Holistic	Individualized
Outcomes	Fatigue/energy levels (100%)	Anxiety (100%)	Patient provider relation (100%)	Peace (71%)	Quality of life (100%)	Goal attainment (95%)
	Sleep quality/duration (95%)	Stress (100%)	Social support (81%)	Spiritual well-being (57%)	Overall well-being (95%)	Patient identified concerns/problems (95%)
	Physical function (85%)	Pain (95%)	Social function (71%)	Meaning in life (52%)	Health status (90%)	Healing (5%)
	Appetite (70%)	Depression (95%)		Serenity (52%)	Life satisfaction (76%)	Vocational (5%)
	Hot flash/night sweats (67%)	Mood (90%)		Spirituality in everyday life (38%)	^a^Prevention of illness (5%)	
	Nausea (55%)	Mindfulness (76%)		Exceptional experiences (35%)		
	^a^Pain (14%)	Empathy (76%)		Spiritual transformation (24%)		
	^a^Disease progression (cancer) (5%)	Adjustment to illness (76%)		Religiosity – beliefs/practices (15%)		
	^a^Dermatological ailment (5%)	Self-esteem (71%)		^a^Mind-body connection (5%)		
	^a^Toxicities (5%)	Disability (71%)				
	^a^Vascular issues (5%)	PTSD (71%)				
	^a^GI function (5%)	Loneliness (67%)				
	^a^Weight issues (5%)	Hopelessness (62%)				
		Distress (62%)				
		Resilience (57%)				
		^a^Memory and brain function (5%)				

^a^Outcomes included in the “other” category by participants

All clinics reported measuring or evaluating health outcomes. Nine clinics (45%) were definitively engaged in such activities and 12 clinics (57%) indicated they were doing this to some degree. The majority of these evaluations (65%) were based on informal patient reports during assessments. Five clinics (25%) applied measures or forms developed in-house, which were not psychometrically assessed or validated. Three clinics (14%) indicated using standardized measures or survey instruments.

Our comparative analysis indicates that practitioner type mix did not result in differences regarding specialization of the clinic or relevance of the IHC components. In terms of health domains and outcomes, clinics that see a generalized patient population were more likely to report spiritual experiences (*p* = 0.007) as a relevant outcome and address religious practices and beliefs (*p* = 0.04) compared to specialized clinics.

## Discussion

We conducted a survey of IHC clinics across Canada in order to better understand what health outcomes are relevant to those who work in and deliver this model of care, based on their practice experience. We specifically focused on this stakeholder group given their unique position in delivering this type of care, directly interacting patients and health consumers, and shaping what is IHC. Therefore, the perspectives of these stakeholders inform how such models of care should be evaluated in order to truly reflect the outcomes and potential benefits of an IHC approach.

The underlying philosophy of care for IHC underscores that all *health domains* are, or at least should be, taken into consideration in an IHC approach; and yet our findings suggest that this was not the case. For example, the holistic domain, although conceptually at the core of the IHC approach, was identified as not relevant by one clinic. This may be because while the concept of holism or the whole person is theoretically defined, how to operationalize it in practice continues to be challenging. In the Database, there are about three times more instruments available within the physical and psychological domains than are available to capture holistic health outcomes; likely a key limitations in the measurement or assessment of this concept. This is echoed by Stewart-Brown et al. [24] and Hunter et al. [25], who report identifying few if any questionnaires designed to measure the concept of holistic health. Similarly, the domain of “social health”, which encompasses relationships with family, community, and health care practitioners, was not relevant for two of the study sites. Interestingly, the patient-provider relationship, an outcome within this domain, is often emphasized as one of the distinguishing characteristics of the IHC approach. The spirituality domain was identified as least relevant of the health domains. Further spirituality-oriented outcomes were indicated as not applicable by four of clinics (which reflected 19% of the sample). The challenge with spirituality in the health context was also identified by Hunter et al. [25] who found that the topic of spiritual health to be highly contentious by IHC practitioners and considered out of the IHC scope by some of the interviewed participants. Interestingly, although considered integral to one’s health, there appears to be tensions regarding its measurement, or more specifically, which components of spirituality could, or should, be scientifically evaluated [26].

With respect to *health outcomes*, the results suggest a range of outcomes across the health domains relevant to understanding the potential benefits of IHC. This is consistent with findings in the literature, where often a broad range of outcomes were identified as necessary to measure to assess IHC approaches. Yet, as also pointed out by Hunter et al. [27], this sheer number continues to pose a significant challenge to researchers and practitioners aiming to evaluate their services or treatment progress. A potentially novel finding of our results is that five health outcomes were consistently identified as relevant across all sites. These are: fatigue, anxiety, stress, patient-provider relationship, and quality of life. These may represent a grouping of outcomes that capture relevant or expected outcomes resulting from of an IHC approach, irrespective of the specific IHC approach, the patient presentation, goals of care, or reasons for seeking IHC. These five outcomes may therefore be used as a core outcome set for IHC that inform an optimum IHC dataset that should be included in any evaluation, facilitating consistent comparisons across the different IHC approaches and settings. While outcome measures such as those being developed by Ritenbaugh et al. [19] that aim to effectively capture complex shifts in the well-being of patients may eventually provide a comprehensive assessment, this proposed core outcomes set may have utility now and may help inform such tool development.

Questions regarding components of IHC were included in the survey to provide confirmation that the clinics in our sample represented an IHC approach, given that recruitment relied on clinics self-identifying as an IHC clinic or practice. An unexpected finding emerged in that the IHC components were not equally relevant across the clinics. For example, one clinic identified a neutral position to a wellness and disease prevention orientation and three clinics indicated this component as somewhat relevant. The whole person care approach was determined to be not relevant by one clinic. And the combination of CAM and conventional therapies was identified as somewhat relevant at one clinic and neither relevant or not relevant by another clinic. Therefore, our definition of IHC, although supported by the literature, may not reflect practice or how it is conceptualized in clinical work or application; this may also reflect an evolving field. These findings raise several important questions regarding the current conceptualization of IHC: How is the idea of holism or a whole person operationalized within a clinical setting? Is the combining of what is currently categorized as CAM and conventional interventions relevant? Perhaps the integration is of different underlying theories or foundations, and not necessarily the intervention, is what needs to be emphasized or focused on? This is suggested in the seminal article by Bell et al. [18], who argue that IHC is not just about bringing together different approaches but rather a higher order system of care.

### Strengths and limitations

There are several limitations of this study that require attention. The Database was used as the primary source for defining IHC health domains and health outcomes. Although a comprehensive database that is conceptually grounded in the literature, it may not necessarily capture all available information on the subject. However, an ‘other’ category was provided in the survey in order to capture additional domains and/or outcomes. It is difficult to determine if the sample surveyed is reflective of IHC clinics in Canada given the challenges with creating a consistent search using various internet search engines and the variations in IHC conceptualization in research and practical applications. The survey results are also limited by the moderate response rate, based on the recruitment strategy utilized. One of the parameters of our search was multi-professional clinics. Therefore, single practitioner clinics providing an integrative approach were excluded but may provide important insight regarding relevant outcomes. Lastly, the survey only asked about the types of practitioners at the clinic and not the number of practitioners, This limits our ability to determine whether a CAM or conventional orientation was predominant within each study sites.

## Conclusions

This study provides an important and unique contribution to the discussions regarding outcomes of IHC. It also highlights the underlying complexity of evaluating the outcomes of IHC interventions, given the variability in relevance regarding health domains and health outcomes, and, how the concept of IHC is applied in real world clinical settings. Although creating a battery of questionnaires that capture the complete experience and impact of IHC has been pursued and new instruments have been proposed, the core set of five outcomes identified may be useful in clarifying what are core outcomes of an IHC approach and development of more comprehensive measurement tools.

## Additional file

Additional file 1: Health Outcomes of Integrative Health Care Practices Survey. (PDF 430 kb)

## Abbreviations

CAM: Complementary and Alternative Medicine
IHC: Integrative Health Care
