# Supplementary material for: Integrative health care – What are the relevant health outcomes from a practice perspective? A survey
Source: BMC Complement Altern Med. 2017 Dec 22;17:548. doi: 10.1186/s12906-017-2041-4 (PMC5741963; doi:10.1186/s12906-017-2041-4)
Supplement: Additional file 1: — Health Outcomes of Integrative Health Care Practices Survey. (PDF 430 kb) [file 12906_2017_2041_MOESM1_ESM.pdf]

Health Outcomes of Integrative Health/Medicine Practice Survey

Background Information
You are invited to complete the Outcomes of Integrative Health Care/Medicine Practice Survey given your experience of providing health care services within such a context. The questionnaire should take approximately 10 minutes to complete.

If you have any questions about the questionnaire or the study, please contact the study lead researcher: Ania Kania-Richmond at akania@ucalgary.ca

Ethics
The study has been ethically approved by the Conjoint Health Research Ethics Board (CHREB) at the University (Ethics ID: REB14-0495).

Definitions
For the purposes of this study, Integrative Health Care/Medicine is defined as a team-based approach to health care provision that includes the select combination of complementary and alternative (CAM) therapies with conventional medical and/or health care treatments. It may be based on the following components:

Holistic/whole person – the person being treated is regarded as an integrated combination of multiple dimensions – body, mind, spirit and context

Personalized care - it is oriented to the specific needs of the patient

Patient-centered - it acknowledges and incorporates the patient's needs and preferences, and actively engages them in their treatment decision making process

Wellness – it aims to enhance an individual's level of well-being and prevent disease

Acknowledgements:
This study is made possible through funding from the Lotte and John Memorial Hecht Foundation.

---

*1. I agree to complete the survey:
- o Yes
- o No – go to *Survey Exit*

Survey Exit

Thank you for your interest in this study. We understand you have declined to complete the Health Outcomes of Integrative Health Care Practice Survey

2. Please provide us with feedback as to why you opted not to complete the survey (optional)

---

*3. What is the name of your clinic?

4. Do you provide permission for the clinic name to be indicated in any research related reports (e.g. publications, conference presentations)?
- Yes – the clinic name can be indicated in research reports and presentations
- No – the clinic name will remain anonymous

*5. What is your position(s) at the clinic? (Check all that apply):
- Owner (or co-owner)
- Director
- Founder
- Board member
- Practitioner
- Administrative staff
- Other (please specify):

*6. Indicate the health care practitioners providing health care services at your clinic. (Check all that apply):
- Acupuncturist
- Chiropractic Doctor
- Massage Therapist
- Medical Doctor
- Naturopathic Doctor
- Nurse
- Physiotherapist
- Psychologist
- Traditional Chinese Medicine Practitioner/Doctor
- Other (please specify):

*7. Indicate the type(s) of patients that receive care your clinic. (Check all that apply):
- General
- Specialized
- Other (please specify):

*8. Indicate the specialization(s) of your practice. (Check all that apply):

- Gender (women's health, men's health)
- Family planning and fertility
- Pediatrics
- Sports Medicine
- Cancer
- Addiction
- Mental Health
- Obesity
- Other (please specify):

*9. Rate how relevant each of the components of integrative health care/medicine is to your clinic:

	Very relevant	Somewhat relevant	Neutral	Not relevant	Do not know
Combining CAM and conventional interventions	○	○	○	○	○
Whole person care/holistic – *the person being treated is regarded as an integrated combination of multiple dimensions – body, mind, spirit and context*	○	○	○	○	○
Patient centered care – *Patient is directly involved in the decision-making process regarding their care and treatment options*	○	○	○	○	○
Personalized/Individualized health care –*Treatment is specifically tailored to address the patient's unique constitution and needs*	○	○	○	○	○
Emphasis on preventing disease and maintaining well-being	○	○	○	○	○

*10. What health domains are relevant and/or important to the care provided at your clinic? (Check all that apply):

- Physical (e.g. physical function, strength, pain)
- Psychological (e.g. anxiety, stress, depression)
- Social (e.g. relationships; work relationships)
- Spiritual (e.g. beliefs, meaning, religious practices
- Overall or global well-being
- Other (please specify):

*11. Which PHYSICAL patient related outcomes are relevant and/or important at your clinic? (Check all that apply):

- Appetite
- Fatigue/energy levels
- Hot flush/night sweats
- Nausea
- Sleep quality/duration
- Not applicable
- Other (please specify):

*12. Which PSYCHOLOGICAL patient related outcomes are relevant and/or important at your clinic? (Check all that apply):

- Pain
- Anxiety
- Stress
- Distress
- Resilience
- Post-traumatic stress disorder (PTSD)
- Depression
- Empathy
- Mood
- Hopelessness
- Adjustment to illness
- Loneliness
- Mindfulness
- Self esteem
- Not applicable
- Other (please specify):

*13. Which SOCIAL patient related outcomes are relevant and/or important at your clinic?

- Quality of relationships/social networks
- Social function
- Social support
- Social interactions
- Patient-provider relationship
- Not applicable
- Other (please specify):

*14. Which SPIRITUAL patient related outcomes are relevant and/or important at your clinic? (Check all that apply):

- Exceptional experiences
- Meaning (in life)
- Peace
- Religiousness – beliefs, practices
- Spiritual well-being
- Level/degree of spirituality in everyday life
- Serenity
- Spiritual transformation
- Not applicable
- Other (please specify):

*15. Which INDIVIDUALIZED patient related outcomes are relevant and/or important at your clinic? (Check all that apply):

- Goal attainment
- Patient identified concerns or problems
- Not applicable
- Other (please specify):

*16. Which GLOBAL or HOLISTIC patient related outcomes are relevant and/or important at your clinic? (Check all that apply):

- Quality of Life
- Overall well-being
- (Overall) health status
- Life satisfaction
- Not applicable
- Other (please specify):

*17. Are you currently measuring or evaluating patient-related outcomes in your clinic?

- Yes
- Somewhat
- No
- Do not know

*18. How do you collect outcomes information/data in your practice? (Check all that apply):

- Clinic developed patient measures/forms (not psychometrically assessed)
- Informal patient reports during assessment (e.g. questions posed by practitioner during a consult)
- Use of standardized measures/forms (e.g. McGill Pain Questionnaire, SF-36)

*19. Are there plans to collect and/or evaluate health outcomes at your clinic in the future?

- Yes
- Somewhat
- Not sure
- No
- Do not know

20. Are there any additional thoughts or comments you would like to share regarding patient related outcomes at your clinic?

*21. Can we contact you as a follow up to this survey to obtain further information regarding health outcomes at your clinic? Please note: any personal information you provide will remain confidential.

- No
- Yes

22. If yes, please provide your preferred method for contact (email, phone number)
